# Exploring disadvantageous inequality aversion in children: how cost and discrepancy influence decision-making

**DOI:** 10.3389/fpsyg.2014.01088

**Published:** 2014-09-26

**Authors:** Amanda Williams, Chris Moore

**Affiliations:** Department of Psychology and Neuroscience, Dalhousie UniversityHalifax, NS, Canada

**Keywords:** social development, inequality aversion, resource allocation

## Abstract

This research examined disadvantageous inequality aversion in 4- and 6-year-old children. Using the resource allocation paradigm, we explored how inequality aversion was influenced by whether a cost was associated with the equitable choice. We also investigated whether preferences for equality differed depending on whether the inequitable choice presented a small or large discrepancy between the payoff of the participant and their partner. The results demonstrated that cost plays a large role in decision-making, as children preferred equality more when there was no cost associated with it compared to when there was a cost. Interestingly, the effect of cost also affected discrepancy, with children more likely to choose equality when the discrepancy was large as opposed to small, in cost trials but not in no cost trials. Finally, the effect of discrepancy also interacted with age, with older children being more sensitive to the discrepancy between themselves and their partner. Together, these results suggest that children’s behavior is not indiscriminately guided by a generalized aversion to inequality or established fairness norms. Alternate motives for inequality aversion are discussed.

## INTRODUCTION

A concern for fairness is important in motivating human cooperation and prosocial behavior. By understanding how this concern emerges in development, we may be better able to support and encourage the development of important social behaviors. Children appear to be sensitive to fairness from a very young age; for example, children as young as 15 months of age will look longer at an unfair distribution of reward than a fair distribution (Sommerville et al., 2012). Young children also demonstrate a sensitivity to inequality in resource distribution situations in which they are one of the recipients. It is now well documented that children begin to share resources early in the preschool period (e.g., Damon, 1975; Rheingold et al., 1976; Eisenberg and Fabes, 1998). When given the opportunity to share resources with others to establish an equal distribution, children will often do so even when a material cost to themselves is required (Thompson et al., 1997; Fehr et al., 2008; Moore, 2009). By 3-years of age, children will also object when a peer or partner receives more than them (LoBue et al., 2011). However, whether children are motivated by fairness concerns in such situations remains unclear. Alternatively, children may be motivated by prosociality in situations where they can forgo a reward in order to deliver a benefit to a partner or by envy resulting from social comparison in situations where they can act to prevent another receiving more than themselves (Shaw and Olson, 2012). The present study examines possible motivations underlying children’s resource allocation, particularly in situations in which they are potentially at a disadvantage compared to a partner. Before elaborating on the particular approach used in the current study, we first briefly describe related work on children’s decision-making in such situations.

Situations in which children are asked to react to an inequitable distribution of resources that favors the partner are said to involve “disadvantageous inequality” (DI). In contrast to advantageous inequality (AI) situations in which an inequitable distribution favors the child, DI situations have received less attention in the literature. However DI situations offer an interesting case for comparing differing motivations underlying fairness. When children show preference for an equal distribution of resources rather than allowing a partner to have more, they may be motivated by a desire for fairness but alternatively they may be motivated by envy resulting from social comparison (Shaw and Olson, 2012). While assessing fairness requires a comparison in the sense that one must compare one’s own resources to the partner’s, in the current study, as in Shaw and Olson’s (2012), “social comparison” refers to the desire to not have less than a partner.

In order to study inequality aversion in a way that eliminated social comparison as a potential motive, Shaw and Olson (2012) used a third party design in which 3- to 8-year-old children decided how to allocate resources to two unknown participants. They found that even younger participants would discard an extra resource when asked to split an uneven amount of resources between two recipients. These results revealed a principle of inequality aversion governing children’s decisions in third party situations, but cannot inform us about how such concerns may operate when children’s own interests are at stake. We know that children as young as 3–4 years of age understand fairness norms, and will report that resources should be split equally, however, it is not until age 7–8 that their sharing behavior aligns with the norms of fairness they endorse (Smith et al., 2013).

Research on DI aversion when children’s own interests are at stake has largely been carried out to examine the origin and development of DI aversion in children and much of it has compared children’s reactions to DI and AI situations (e.g., Fehr et al., 2008; Blake and McAuliffe, 2011; LoBue et al., 2011). In general, this work shows that aversion to these two forms of inequality develops along distinct developmental trajectories, with children demonstrating a dislike for inequality that disadvantages themselves several years before they exhibit aversion toward inequality that favors themselves. For example, LoBue et al. (2011) found that children as young as 3 years of age would object when an experimenter distributed resources in a way that disadvantaged themselves in comparison to a partner. However, children were less likely to object to unequal distributions that placed them at an advantage in comparison to their partner. This finding suggests that children’s motives in DI situations are at least in part motivated by envy resulting from negative social comparison.

In the study by LoBue et al. (2011), children responded to unfair resource distributions imposed by an adult. However, when children have the opportunity to decide themselves how resources are distributed across self and a partner, there is also evidence that children will avoid DI. In perhaps the first experiment on DI situations in children, Fehr et al. (2008) used a forced choice resource allocation task to introduce an “envy” decision in which 3- to 8-year-olds chose between an equal distribution of reward (one candy for both self and partner) and an unequal distribution of reward that disadvantaged themselves (one candy for self and two for the partner). Equitable choices in this DI trial were compared with two AI trials, in which equality came with either a cost or no cost. Though preferences for equality differed across trials, an overall increase in equitable decisions with age was observed, and the authors cast this development in terms of a principle of inequality aversion general to both AI and DI situations. There was, however, no direct evidence that the same concerns were motivating decision-making in the different trials types. The increase in equitable choices observed in the DI choices is particularly ambiguous because the level of preference for the equal choice at the younger age was no different from chance. Because the DI choice did not involve a cost, it is entirely possible that the younger children were only paying attention to their own reward and were unaffected by the disadvantageous comparison between their reward and those of their partner. Without a condition in which avoiding DI comes at a cost, it is not possible to determine whether these younger children really are avoiding inequality, or what, if any, motive they have for doing so.

Subsequent work has shown that a preference for equality sometimes presents itself even when there is a cost associated with removing the comparative disadvantage. Blake and McAuliffe (2011) presented 4- to 8-year-olds with an unequal number of candies for themselves and a partner, and asked them if they would like to accept or reject the offer (in which case neither party received anything). In DI trials children were offered one candy for themselves, and four for their partner, while in AI trials children were offered four candies for self and one for partner. While children did not show inequality aversion to AI until 8 years of age, children across all age groups commonly rejected DI offers. As in the case of LoBue et al. (2011) discussed earlier, the different developmental patterns suggest that avoidance of AI and DI are differentially motivated at least in young children (Blake and McAuliffe, 2011). The results also suggest that when the other stands to get a much larger reward than the self, children are strongly motivated to reject the resource allocation.

The two studies just described remain the only two that have directly examined children’s self-involved DI decisions in resource allocation contexts across different age groups. However, comparison across the two studies is difficult because they differed in two key aspects. Fehr et al. (2008) presented DI choices for which there was no cost to making the equitable choice (the children received the same reward either way) and the potential discrepancy between self and partner was relatively small (one vs. two). In contrast, Blake and McAuliffe (2011) presented choices for which there was a cost to avoiding DI (both participants lost everything), and the potential inequality was relatively large (one vs. four). It is conceivable that both of these variables have an impact on children’s decisions in DI contexts. Younger children may have a tendency to focus on their own reward exclusively, and therefore a cost choice could lead to a lower level of inequality aversion compared to a no cost choice, for which children may choose essentially randomly. The size of the discrepancy between self and other may also have an effect in that the larger discrepancy, the greater the potential for a negative social comparison and resultant feelings of envy. So, if envy is motivating decisions in DI situations, children may avoid inequality to a greater extent when the discrepancy is large compared to when it is small.

To generate a clearer picture of how young children’s decisions in DI situations are motivated, we presented 4- and 6-year-old children with a series of decisions, each involving a choice between an equal distribution of resources and an unequal distribution that favored the partner. We varied both the cost of making an equitable decision and the size of the discrepancy between the reward for self and other in the DI case. First, we compared the type of DI trial introduced by Fehr et al. (2008) in which there was no cost to the participant for either choice, with a costly trial type in which the child would have to give up their own resource to avoid inequality (cf. Blake and McAuliffe, 2011). Although how cost influences DI has not been systematically explored, cost has been shown to influence behavior in other social contexts. In situations of AI, children demonstrate weaker preferences for equality when it comes with a cost (Thompson et al., 1997; Fehr et al., 2008; Moore, 2009). Children also judge others less harshly for not helping someone in need when there are high costs associated with helping, compared to when costs are low (Sierksma et al., 2014). Given these established cost effects across other social domains, it was hypothesized that cost would also influence decision-making in situations of DI. Specifically, it was expected that children would show a stronger preference for equality when there was no cost associated with it, partially because those children who only paid attention to their own payoff would be more likely to choose the equal option. While the absence of a cost effect would provide support for inequality aversion motives, an effect of cost would suggest children’s decision making is influenced by what is in their own best interest, as opposed to fairness norms.

Second, we compared children’s decisions in DI situations involving two different discrepancies between the participant’s and the other recipient’s resources in the unequal option. In half the trials the discrepancy was small (one for self; two for partner) and in half the trials the discrepancy was larger (one for self; five for partner). The reasoning here was that if children are primarily concerned with maintaining equality, in accordance with fairness norms, then there should be little or no difference between egalitarian choices in these two trial types. However, if they are responding more to the envy engendered by social comparison between self and other, then the larger the discrepancy, the more they may be inclined to reject it. Therefore, in line with the idea that children’s decisions in DI situations may be motivated by social comparison and envy concerns, we predicted more egalitarian choices would be made in large discrepancy trials compared to small discrepancy trials.

To summarize, combining these two variables yielded four types of trials: no cost with a small discrepancy (1,1 vs. 1,2); no cost with a large discrepancy (1,1 vs. 1,5); cost with a small discrepancy (0,0 vs. 1,2); and cost with a large discrepancy (0,0 vs. 1,5). Children of 4 and 6 years of age were tested because evidence of increasing inequality aversion in the envy trial type has been observed in this age range (e.g., Fehr et al., 2008), but previous research has not adequately explored motives underlying decision-making in DI situations in children of these ages. Given that inequality aversion has been observed to increase with age in multiple resource allocation situations (e.g, Fehr et al., 2008; Blake and McAuliffe, 2011; Shaw and Olson, 2012) it was predicated that older children would make more egalitarian decisions compared to younger children. In view of the limited background literature on DI, no specific predictions were made regarding interactions between age, cost and discrepancy.

## MATERIALS AND METHODS

### PARTICIPANTS

Forty-two typically developing children drawn from a predominately white middle-class neighborhood in a small Canadian city participated in this study, which was approved by the University’s research ethics board. Participants were recruited from a database, as well as a variety of community classes and events. Two participants were excluded due to incomplete participation leaving a sample of 40 children. The 4-year-old group (10 males, 10 females) had a mean age of 52 months, 6 days (ranging from 42 months, 17 days to 57 months, 2 days). The 6-year-old group (8 females, 12 males), had a mean age of 75 months, 29 days (ranging from 68 months, 6 days to 82 months, 24 days).

### PROCEDURE

All testing took place in the lab, and began once parental consent and participant assent was obtained. Following the approach introduced by Moore (2009), children were asked to think of, and name a friend they enjoyed playing with. Children were then asked to draw themselves and their friend from memory on individual 4′′ by 6′′ inch blank cards. Before testing started children were asked to identify their drawings, and were corrected if either drawing was misremembered.

The researcher then faced the child and said, “*We’re going to play a choosing game. In this game, sometimes you might choose stickers for you and (friend’s name) and sometimes you might choose not to take any stickers. The stickers you choose for yourself will go here, and the stickers you choose for (friend’s name) will go here.”*

Brightly colored stickers portraying popular television characters that children found attractive, and appealing, were used as the resource. A variety of different stickers was used with each participant to ensure that the stickers remained salient and attractive reward throughout the duration of the task. Children were given a sticker book to place stickers they chose for themselves, and stickers chosen for their friend were placed in a paper bag.

**FIGURE 1:**
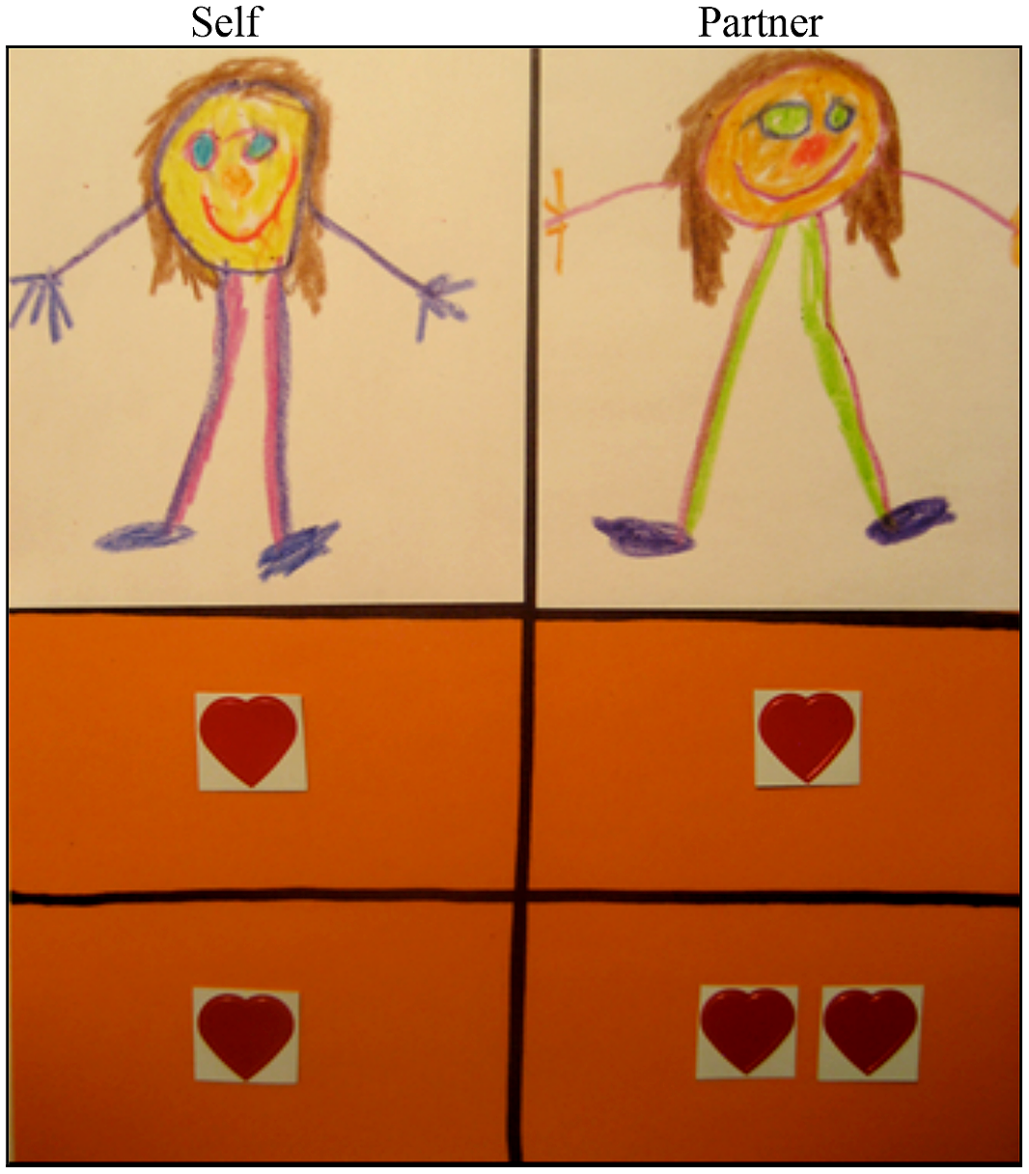
**Method of trial presentation showing the small discrepancy, no cost trial type**.

Before the test trials began, each child participated in one practice trial (choosing between one or two stickers for themselves) to familiarize them with the format of the game. Responses were recorded but not analyzed. There were four trial types and children participated in three trials of each, for a total of 12 test trials. Trials were presented in three blocks. Each block contained one of each of the four different trials types. The order of the trial types was varied within block, and the order of the blocks was varied across participants to ensure no order effects contributed to the findings. In each trial the picture of the participant and their partner were placed on a piece of paper, and the two alternative distributions were laid out below each picture, and divided by a line (see **Figure 1**). Children were told, “*Here you are and here is (partner’s name).”*

In each trial children were asked “*Would you like to choose (n) sticker(s) for yourself, and (n) for (friend’s name), or would you like to choose (n) sticker(s) for yourself and (n) for (friend’s name)?”* In cost trials the choices were (0,0 vs. 1,2) in SD trials, and (0,0 vs. 1,5) in LD trials. In no cost trials the choices were (1,1 vs. 1,2) in SD trials, and (1,1, vs. 1,5) in LD trials. Participation for each child lasted approximately 15 min. Each session for which parental consent to videotape was obtained was recorded for verification and coding purposes.

## RESULTS

**FIGURE 2:**
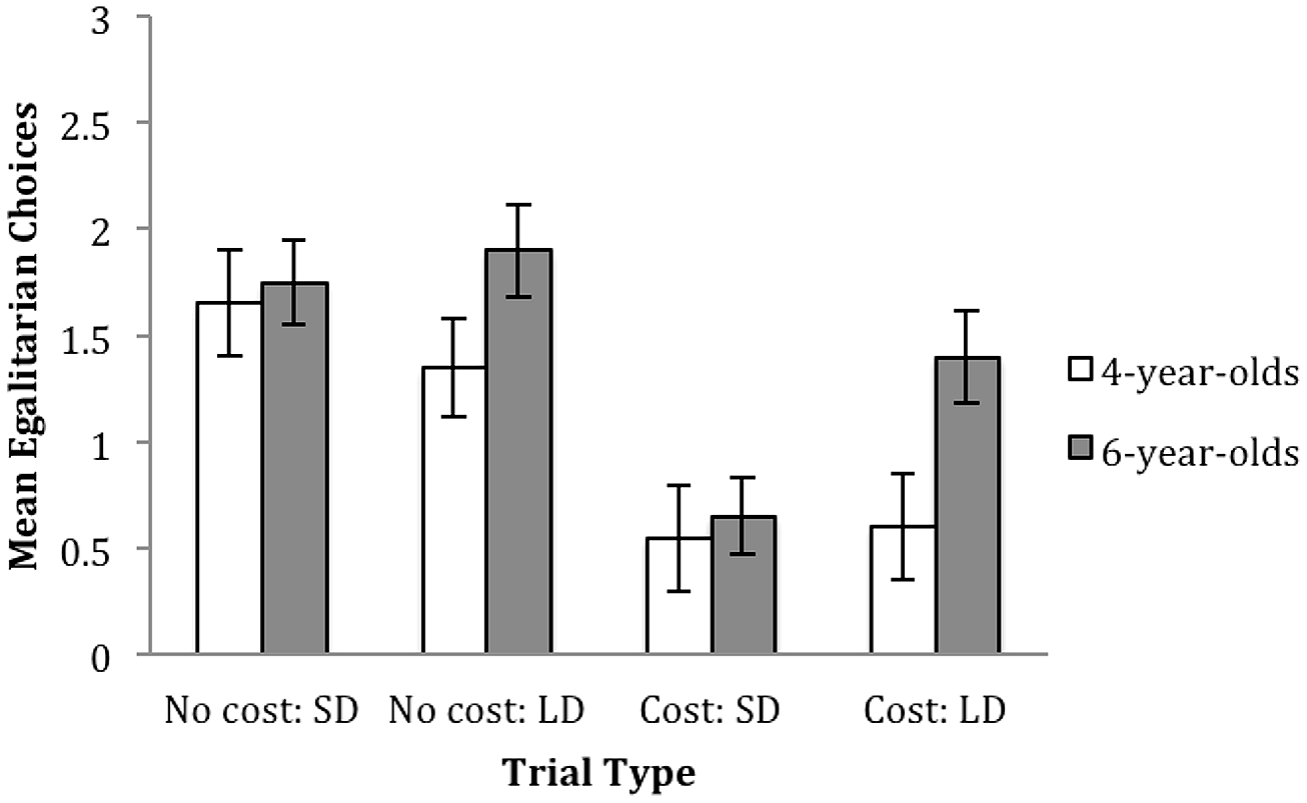
**Average egalitarian decisions (with standard error bars), made by 4 and 6-year-old children in no cost and cost trials with small and large discrepancies**.

Children received one point for each egalitarian choice made (0,0 in cost trials and 1,1 in no cost trials), therefore receiving an overall score ranging from “0” to “3” for each trial type. Descriptive statistics can be seen in **Figure 2**.

A 2 × 2 × 2 mixed model repeated measures ANOVA with cost (cost, no cost) and discrepancy (SD, LD) as within subject factors, and age as a between subjects factor was performed with the number of egalitarian choices as the dependent variable. Between subjects, no significant main effect of age was observed, *F*(1,38) = 2.410, *p* = 0.129, $\eta_{p}^{2}$ = 0.060. There was a significant main effect of cost, *F*(1,38) = 37.272, *p* < 0.001, $\eta_{p}^{2}$ = 0.495, with more egalitarian decisions overall in no cost trials (*M* = 3.33, SD = 1.64) compared to cost trials (*M* = 1.6, SD = 2.01). There was no significant interaction between cost and age, *F*(1,38) = 0.196, *p* = 0.661, $\eta_{p}^{2}$ = 0.005.

Although there was no significant main effect of discrepancy, *F*(1,38) = 2.018, *p* = 0.164, $\eta_{p}^{2}$ = 0.050, and no significant three-way interaction between cost, discrepancy, and age, *F*(1,38) = 0.400, *p* = 0.531, $\eta_{p}^{2}$ = 0.010, two significant interactions involving discrepancy emerged. There were significant interactions between cost and discrepancy, *F*(1,38) = 5.778, *p* = 0.021, $\eta_{p}^{2}$ = 0.132, and between discrepancy and age, *F*(1,38) = 6.317, *p* = 0.016, $\eta_{p}^{2}$ = 0.143. These interactions were explored using follow-up paired samples *t*-tests.

To follow up the interaction of cost and discrepancy, paired *t*-tests showed that for cost trials children were more likely to choose the egalitarian option when the discrepancy was large (*M* = 1.0, SD = 1.13) than when it was small (*M* = 0.6, SD = 0.98), *t*(39) = -3.766, *p* = 0.001, but there was no difference between the large (*M* = 1.63, SD = 1.03) and small (*M* = 1.7, SD = 1.01) discrepancy for no cost trials, *t*(39) = 0.386, *p* = 0.701. In line with the main effect of cost, children preferred equality more in no cost, compared to cost trials in both small discrepancy, *t*(39) = –6.169, *p* < 0.001, and large discrepancy trials, *t*(39) = -3.838, *p* < 0.001.

To examine the interaction involving age, the discrepancy effect was examined for each age. It was found that the younger (4-year-old) group showed no significant effect of discrepancy, *t*(19) = 0.925, *p* = 0.367, choosing the egalitarian option with equal frequency whether the discrepancy was small (*M* = 2.2, SD = 1.88) or large (*M* = 1.95, SD = 1.99). In contrast, there was a significant effect of discrepancy for the 6-year-olds, *t*(19) = -2.438, *p* = 0.025, who chose the egalitarian option more often when the discrepancy was large (*M* = 3.3, SD = 1.59) compared to when it was small (*M* = 2.4, SD = 1.43).

Independent samples *t*-tests were run comparing 4-year-olds, and 6-year-olds preferences in small, and large discrepancy trials. While no differences between 4 and 6-year-olds were observed in small discrepancy trials, *t*(38) = -0.379, *p* = 0.707, a significant difference was observed in large discrepancy trials, *t*(38) = -2.371, *p* = 0.023, with 6-year-olds choosing the equitable option more often than 4-year-olds.

## DISCUSSION

The goal of the current study was to explore how cost, discrepancy, and age influenced young children’s decision-making in DI situations, and to gain insight as to whether inequality aversion or social comparison was motivating their behavior. Four- and 6-year-old children were presented with resource allocation choices in which one option delivered a greater benefit to a friend, and the other option was egalitarian. Across trials, egalitarian choices entailed either a cost to the children’s own payoff, or no cost. Trials also differed in terms of the discrepancy between the resources available to self and other in the inequitable option, yielding four distinct trial types; small discrepancy no cost trials (1,1 vs. 1,2), large discrepancy no cost trials (1,1 vs. 1,5), small discrepancy cost trials (0,0 vs. 1,2), and large discrepancy cost trials (0,0 vs. 1,5). We expected that children would prefer equality more when there was no cost associated with it, and that older children would demonstrate a stronger aversion to inequality. It was proposed that if a generalized aversion to inequality or fairness norms motivated decision-making, discrepancy would not influence preferences for equality. However, if social comparison was influencing decision-making, children would show a stronger preference for equality in LD trials compared to SD trials. The findings demonstrated that both cost and discrepancy influenced children’s decisions. Therefore there does not appear to be a simple or undifferentiated aversion to inequality operating in these children. Here we discuss the key results in more detail, and offer an account of the development of inequality aversion in DI situations.

The results demonstrated that as hypothesized, children preferred equality more in no cost trials compared to cost trials; more often choosing to prevent their partner from receiving a larger reward when they were not required to sacrifice their own reward to do so. This finding was consistent in both SD and LD trials, and suggests that an important determinant of children’s decisions in DI situations is whether a sacrifice is needed to achieve equality. Like Fehr et al. (2008), we found that when there was no cost to the egalitarian choice children chose this option over 50% of the time, and there was no strong difference between 4- and 6-year-olds to act in this way. However, we found that when there was a cost to the egalitarian choice, and children had to sacrifice their resources, this option was chosen much less frequently. Equality alone is therefore not the issue for these children; if equality comes at a cost it will be largely forgone.

Nevertheless, our results do not suggest that children are completely unwilling to pay a cost to avoid DI. Whereas no overall effect of discrepancy was observed, the effect of cost was influenced by the size of the discrepancy between the resources for the child and their friend. Discrepancy did not influence decision-making in no cost trials, however in costly trials children were more likely to choose the egalitarian option when the discrepancy was large compared to when it was small. This suggests that in cost trials, social comparison was influencing decision-making. Although Blake and McAuliffe (2011) did not explore different discrepancies, our observation in large discrepancy cost trials is consistent with their claim that 4–8 year-olds will sacrifice resources to prevent DI in which the other received four times as many resources. Our results extend theirs in showing that the size of the discrepancy makes a difference to children’s tendency to pay the cost of preventing DI – children are more likely to pay to avoid a large discrepancy, compared to a small discrepancy. However, a single motivation based on social comparison cannot explain preferences for equality across all decisions as there was no overall effect of discrepancy and in particular no effect of discrepancy in no cost trials. Interestingly, research suggests that when costs are low children perceive prosociality as morally obligated, while in costly situations they may take other factors into consideration (Sierksma et al., 2014). Therefore, it could be that when there is no cost associated with equality choosing the equitable decision is an easy or even default decision regardless of discrepancy. However, when a cost is associated with equality children may be more sensitive to other considerations such as the comparison between themselves and their partner making a larger discrepancy more likely to motivate a sacrifice. This could explain why a discrepancy effect was observed in cost trials, but no overall effect of discrepancy was observed.

Finally, although no overall age effect was observed, the interaction between age and discrepancy provided evidence of a developmental change in the conditions under which children seek to prevent DI. It was argued that an increase in equitable decisions corresponding with a larger discrepancy would provide support for social comparison motives. An effect of discrepancy was indeed observed but only for the older children. The 4-year-old children’s preferences for equality did not differ depending on whether the discrepancy between their own resources and their partner’s was small or large. This pattern of behavior is entirely consistent with a simpler account of their decision-making: younger children were only paying attention to their own payoff, and ignoring the payoff for their partner. Thus, in no cost trials where both options resulted in one sticker for the self, 4-year-olds chose each option in about half the trials no matter what the reward conferred to the other was. In cost trials where one option resulted in a smaller reward, they made the more rewarding choice on the large majority of trials, again regardless of the other’s payoff. Therefore, it is likely that social comparison and envy played little or no role for these children.

The 6-year-olds showed a different pattern of choices. They were significantly more likely than younger children to avoid DI in the large discrepancy trials. Clearly they were more reluctant than the younger children to let their friend have many more resources than them, although they showed similar equanimity to the younger children when the discrepancy between self and friend was small. The older children therefore, were displaying an aversion to the large discrepancy between own and other’s resources, but because this aversion did not extend similarly to the small discrepancy trials it appeared not to reflect a general inequality aversion or fairness norm. Therefore, it seems that for older children the large discrepancy led to a more negative social comparison, and subsequently increased associated feelings of envy.

Age related changes have previously been documented in DI resource allocation contexts (Fehr et al., 2008; Blake and McAuliffe, 2011; Shaw and Olson, 2012). However, earlier studies have not systematically manipulated different aspects of the DI decisions. If we are correct that different processes are underlying the decisions at different ages in the current experiment, then this would explain why, with different variables manipulated, we did not observe an overall main effect of age. The number of equitable choices made in some trial types may not have differed across age groups, but it is possible that the processes underlying these choices differed from those underlying other trial types. For example, the fact that no overall age effect was observed could be partly due to the robust cost effect that was consistent across both age groups. As evidence of a more generalized aversion to inequality has been observed in older children’s decision making (e.g., Blake and McAuliffe, 2011; Shaw and Olson, 2012) it could be the case that social comparison continues to play a role in making fairness evaluations, but children become better able to overcome being influenced by negative feelings with age.

One limitation of the current study is that a variety of different stickers was used for each child and there was no pretest to determine how much each child liked the various stickers. This approach was taken to ensure that the stickers remained novel and attractive over the course of the testing. However, it is possible that the children may have found some stickers more attractive than others, and this variability might have influenced the results, although not in a systematic way. It should also be noted that the inferences from the current study are limited in that the children made their choices with a friend as the recipient, and these results may not generalize to other partners outside of the context of a friendship. It is possible that using friends as partners could have produced more variability in terms of the nature of the relationship between the children and their partners than would have been observed had we used anonymous or unknown partners. As friends have been shown to elicit more generous behavior (e.g., Moore, 2009) it is possible that with a different partner less prosociality would have been observed. Future research would benefit from exploring how preferences for equality in situations of DI differ depending on whether a partner is known or unknown, or a friend or non-friend (cf. Moore, 2009, for AI situations). Future research should also further investigate factors that may influence preferences for equality in DI situations, and the motivations behind such preferences. Exploring how discrepancy influences decision-making in older age groups, as well as the inclusion of additional measures (for example, asking children to explain the reasoning behind their decisions) could help shed more light on how motivations underlying decision-making change throughout development.

In summary, we found no evidence of generalized inequality aversion in 4- and 6-year-olds’ decisions in DI situations. Most obviously, cost and no cost choices elicited different levels of egalitarian choices, with children preferring equality more when there was no cost associated with it. In cost trials discrepancy also played a role, as children were more likely to sacrifice their own resources to prevent their partner from receiving many more stickers than them, as opposed to just one more. Further, the finding that 6-year-olds choose the equitable option more in LD trials compared to the 4-year-olds suggests that children at this age may be particularly sensitive to social comparison, and their desire for equality may be more influenced by social comparison, as opposed to a more generalized aversion to inequality. Taken together, our findings suggest that between 4 and 6 years children become more attuned to the social comparison between self and other when allocating resources in potentially DI situations. Whereas 4-year-olds appear to want to maintain a degree of equality between self and other, they are not willing to pay for it. This pattern can be characterized perhaps as a weak inequality aversion in that equality is preferred when nothing is at stake personally (cf, Shaw and Olson, 2012). By 6 years, children are sensitive to the social comparison such that a desire for equality is increased in accordance with possible size of the negative comparison and even if there is a cost. Interestingly, this age difference is inconsistent with an increasing adherence with age to a social norm of fairness, as the older children showed even less “normative” behavior than the younger children. So, although children do seem to reach a point at about 8 years where their resource allocation decisions are organized in relation to a fairness norm (Fehr et al., 2008; Blake and McAuliffe, 2011; Shaw and Olson, 2012), it appears they first undergo a developmental shift that makes them more prone to social comparison and envy. It is even possible that this shift is a necessary stage in the development of more normative behavior. Social comparison may set up the motivational conditions for fairness, and while DI situations may elicit envy, AI situations may elicit social welfare concerns such as altruism (Shaw and Olson, 2012). The resolution of these incompatible experiences resulting from inequality situations may come, with appropriate cultural support, through an adherence to a more general norm of equality.

## Conflict of Interest Statement

The Associate Editor, Dr. Markus Paulus, declares that despite having collaborated with author Dr. Chris Moore, the review process was handled objectively and no conflict of interest exists. The authors declare that the research was conducted in the absence of any commercial or financial relationships that could be construed as a potential conflict of interest.
